# Evaluation of the safety and immunogenicity of the oral inactivated multivalent enterotoxigenic *Escherichia coli* vaccine ETVAX in Bangladeshi adults in a double-blind, randomized, placebo-controlled Phase I trial using electrochemiluminescence and ELISA assays for immunogenicity analyses

**DOI:** 10.1016/j.vaccine.2018.11.040

**Published:** 2019-09-03

**Authors:** Marjahan Akhtar, Mohiul I. Chowdhury, Taufiqur R. Bhuiyan, Joanna Kaim, Tasnuva Ahmed, Tanzeem A. Rafique, Arifuzzaman Khan, Sadia I.A. Rahman, Farhana Khanam, Yasmin A. Begum, Mir Z. Sharif, Laila N. Islam, Nils Carlin, Nicole Maier, Alan Fix, Thomas F. Wierzba, Richard I. Walker, A. Louis Bourgeois, Ann-Mari Svennerholm, Firdausi Qadri, Anna Lundgren

**Affiliations:** a icddr,b (International Centre for Diarrhoeal Disease Research, Bangladesh), Dhaka, Bangladesh; b GUVAX (Gothenburg University Vaccine Research Institute), Dept. of Microbiology and Immunology, Inst. of Biomedicine, University of Gothenburg, Sweden; c Scandinavian Biopharma, Solna, Sweden; d PATH, Washington, DC, USA; e Dept. of Biochemistry and Molecular Biology, University of Dhaka, Bangladesh

**Keywords:** ETEC, Vaccine, Antibodies in lymphocyte supernatant, Antibody-secreting cell, IgA, Adult, ELISA, Electrochemiluminescence, AEs, adverse events, ALS, antibodies in lymphocyte supernatant, ASC, antibody-secreting cell, CFs, colonization factors, CTB, cholera toxin B-subunit, dmLT, double mutant heat labile toxin, ECL, electrochemiluminescence, ETEC, enterotoxigenic *Escherichia coli*, LT, heat labile toxin, LCTB*A*, CTB/LTB hybrid protein, MSD, Meso Scale Discovery, PBMC, peripheral blood mononuclear cells, SAE, serious adverse event, SIgA, secretory IgA, ST, heat-stable toxin

## Abstract

The safety and immunogenicity of the second generation oral enterotoxigenic *Escherichia coli* (ETEC) vaccine ETVAX, consisting of inactivated recombinant *E. coli* strains over-expressing the colonization factors (CFs) CFA/I, CS3, CS5 and CS6 and the heat labile toxoid LCTB*A,* were evaluated in Bangladeshi volunteers. To enable analysis of antibody responses against multiple vaccine antigens for subsequent use in small sample volumes from children, a sensitive electrochemiluminescence (ECL) assay for analysis of intestine-derived antibody-secreting cell responses using the antibodies in lymphocyte secretions (ALS) assay was established using Meso Scale Discovery technology.

Three groups of Bangladeshi adults (n = 15 per group) received two oral doses of ETVAX with or without double mutant LT (dmLT) adjuvant or placebo in the initial part of a randomized, double-blind, placebo-controlled, age-descending, dose-escalation trial. CF- and LTB-specific ALS and plasma IgA responses were analyzed by ECL and/or ELISA.

ETVAX was safe and well tolerated in the adults. Magnitudes of IgA ALS responses determined by ECL and ELISA correlated well (r = 0.85 to 0.98 for the five primary antigens, *P* < 0.001) and ECL was selected as the ALS readout method. ALS IgA responses against each of the primary antigens were detected in 87–100% of vaccinees after the first and in 100% after the second vaccine dose. Plasma IgA responses against different CFs and LTB were observed in 62–93% and 100% of vaccinees, respectively. No statistically significant adjuvant effect of dmLT on antibody responses to any antigen was detected, but the overall antigenic breadth of the plasma IgA response tended to favor the adjuvanted vaccine when responses to 4 or more or 5 vaccine antigens were considered. Responses in placebo recipients were infrequent and mainly detected against single antigens.

The promising results in adults supported testing ETVAX in descending age groups of children.

ClinicalTrials.gov Identifier: NCT02531802.

## Highlights

• The killed oral ETEC vaccine ETVAX ± dmLT adjuvant was safe in Bangladeshi adults.
• All vaccinees responded to all 5 primary vaccine antigens in ALS specimens.
• A majority of vaccinees responded to ≥4 antigens in plasma specimens.
• A sensitive electrochemiluminescence assay was established for small sample volumes.
• ALS responses measured by electrochemiluminescence and ELISA assays correlated well.

## 1 Introduction

Enterotoxigenic *Escherichia coli* (ETEC) remains one of the most common bacterial pathogens causing diarrhea in children as well as adults in developing countries [1], [2], [3]. ETEC colonize the intestinal mucosa by different colonization factors (CFs) and subsequently release heat labile (LT) and/or heat stable (ST) enterotoxins causing diarrhea [4], [5]. Since ETEC do not invade intestinal epithelial cells, immune protection is most likely provided by locally produced secretory IgA (SIgA) antibodies [6]. To achieve broad protection against ETEC, immunity against both LT and CFs would be advantageous; anti-ST immunity is difficult to induce in a safe manner due to the small size of the ST peptide and potential immunological cross-reactivity with human guanylin and uroguanylin [5], [6], [7], [8], [9].

One approach to achieve immunity to ETEC which has been extensively investigated is to immunize orally with killed bacteria expressing common CFs and to administer the vaccine with a LT-like toxoid [6], [7]. A first generation whole cell vaccine, consisting of formalin-inactivated ETEC bacteria expressing CFA/I and CS1 to CS5, combined with cholera toxin B-subunit (CTB), which is highly homologous to LT B-subunit (LTB), was shown to be immunogenic in children and adults in endemic areas and to protect against moderate/severe diarrhea in adult travellers [10], [11], [12]. However, a full dose of the vaccine caused vomiting in 6–17 months old Bangladeshi children; hence fractionated doses were tested and a quarter dose was found to be safe [13]. The vaccine did not confer protection in 6–24 months old Egyptian children, but both vaccinated and unvaccinated children experienced mainly mild disease during the study period and the impact on moderate/severe diarrhea could not be evaluated [7]. Based on these results, an improved second generation multivalent oral ETEC vaccine (ETVAX) was developed, containing inactivated *E. coli* strains over-expressing CFA/I, CS3, CS5 and CS6 antigens at significantly higher levels than the bacteria in the first generation vaccine [14]. In contrast to the first generation vaccine, ETVAX includes the common CF CS6 in an immunogenic form and is administered together with the more LT-like toxoid LCTB*A* [15], [16]*.* To further enhance the immunogenicity of the vaccine, particularly when given at low dosages, the vaccine may be combined with the double mutant LT (dmLT) adjuvant [14], [17]. When tested in Swedish adults, ETVAX with or without dmLT was found to be safe and to induce significant fecal SIgA responses as well as IgA antibody-secreting cell (ASC) responses, as determined by analysis of specific antibodies secreted into the culture supernatants using the antibodies in lymphocyte supernatants (ALS) assay, against all CFs and LTB [16]*.* Addition of 10 µg dmLT to the vaccine significantly enhanced ALS responses to CS6 only [16]. ETVAX also induced long-lasting immunological memory in Swedish adults [18]. Our recent results also demonstrated that ETVAX may induce IgA antibody responses that cross-react with CFs belonging to the same CF families, possibly expanding the coverage of the vaccine [19]. These successful results have led to clinical evaluation of ETVAX in a large phase I/II trial in adults and lower age groups (5 years to 6 months) in Bangladesh. Since limited blood volumes can be collected from children and infants, a new assay for analysis of ALS responses using small sample volumes needed to be established and optimized using samples from adults to allow subsequent analyses in younger age groups.

After infection or vaccination in the intestinal mucosa, activated intestinal lymphocytes transiently migrate to the circulation before homing back to the mucosa. Therefore, ASCs present among peripheral blood mononuclear cells (PBMCs) are suitable surrogate markers of mucosal immunity [13], [20], [21], [22], [23], [24], particularly if blood samples are collected at optimal time points after lymphocyte activation [16], [18], [25], [26]. ASC responses can be analyzed using ELISPOT or by ALS, which is commonly based on ELISA techniques for detection of secreted antibodies in the culture medium, and results from the two assays correlate very well with each other [15], [24], [27], [28]. In clinical trials with multivalent vaccines such as ETVAX, ALS responses to several vaccine antigens are usually evaluated, requiring relatively large volumes of ALS specimens. Hence, to allow analysis of ALS responses to multiple vaccine antigens in children, sensitive and specific procedures are required.

An electrochemiluminescence (ECL) assay may be used as an alternative to conventional colorimetric ELISA methods. ECL-based techniques generally have high sensitivity, good reproducibility, and a wide dynamic analysis range reducing the need for sample titration, and allow smaller sample volumes than ELISA [29], [30], [31]. The Meso Scale Discovery (MSD) technology is an ECL method utilizing sulfo-tag-labelled detection antibodies, which emit light upon electrochemical stimulation via electrodes integrated in the bottom of carbon-based microtiter plates. Soluble antigens bind efficiently to the carbon surface of the wells, without the need for conjugation or chemical antigen modification. MSD-based ECL assays have previously been established for analysis of serum antibodies against different parenteral vaccines and infections, using both single- and multiplex analysis platforms [32], [33], [34], [35], but have, to our knowledge, not previously been reported for analysis of serum antibody or ASC/ALS responses after mucosal vaccination.

The primary objective of the study presented here was to evaluate the safety of ETVAX alone or together with dmLT adjuvant in Bangladeshi adults; the secondary objective was to evaluate the immunogenicity of ETVAX as a basis for studies in Bangladeshi children and infants. The study also included establishment of an ECL assay based on the MSD ECL technology for detection of ALS responses against multiple ETVAX antigens in small sample volumes for subsequent use in studies in younger age groups.

## 2 Materials and methods

### 2.1 Study design

This study was planned as a randomized, double-blinded, placebo-controlled, dose-escalation and age-descending Phase I/II trial conducted in Mirpur, Dhaka, Bangladesh. Healthy adults (Phase I) and children (Phase II, including older children 24–59 months, younger children 12–23 months and infants 6–11 months) were sequentially immunized and safety was confirmed at each dosage level in each age group before the study proceeded to the next stage. The complete study design can be found at ClinicalTrials.cov (Identifier: NCT02531802). Data from the adult part were unblinded before the studies in children were completed, consistent with the study protocol. Data from the adult part of the study is reported here; results from the younger age groups will be reported later.

The study was performed in accordance with the Declaration of Helsinki and approved by the Research Review and Ethical Review Committees of the International Review Board of the International Centre for Diarrhoeal Disease Research, Bangladesh (icddr,b) and the Western Institutional Review Board, USA. The trial was conducted under oversight of the Federal Drug Administration, USA. Informed written consent was obtained from each participant.

### 2.2 Vaccine and dmLT adjuvant

Two doses of the 2nd generation multivalent ETEC vaccine (ETVAX, produced for Scandinavian Biopharma by Biovian Oy, Tykistökatu 6B FI-20520 Turku, Finland, lot BX1003574) was given orally to study participants at two weeks interval. The vaccine consists of 8x10^10^ inactivated *E. coli* bacteria (strains ETEX 21–24, 2 × 10^10^ bacteria/strain) recombinantly induced to express high amounts of CFA/I, CS3, CS5 and CS6 antigens, respectively and mixed with 1 mg of LCTB*A* protein, a recombinantly produced LTB/CTB hybrid protein [14], [16], [36]. This single dose level was administered to adults. A vaccine dose was suspended in 150 ml bicarbonate buffer (Recipharm, Sweden) and given alone or together with 10 µg of the adjuvant dmLT (R192G/L211A, lot 1575, Walter Reed Army Institute, Silver Spring, MD, USA) [17]. Children and infants received lower dosages of vaccine (1/8–1/2 adult dosage) alone or combined with dmLT (2.5–10 µg) or placebo, as described in detail in the Clinical.Trials.gov registry.

### 2.3 Screening and randomization

Healthy adult participants (18–45 years) were screened 4–7 days prior to enrollment for eligibility based on medical history, clinical examination and laboratory tests. Key exclusion criteria included known significant systemic disorders, congenital abdominal disorders, diarrheal or febrile illnesses in the past 7 days, positive pregnancy test and use of antibiotics or immunosuppressant medications within 14 days prior to enrollment. Participants positive for ETEC, *Shigella*, *Vibrio cholerae* or *Salmonella,* as determined by culture of a fecal sample collected during the screening period, were also excluded. All participants were from similar socioeconomic backgrounds. A randomization list with treatment information for each participant was generated by the statistical and data management group at the EMMES Corporation, Rockville, MD, USA and maintained by a pharmacist, who was not otherwise involved in the study.

Enrolled participants were randomized in a double blinded manner into one of three groups (1:1:1): (A) placebo (n = 15, buffer alone), (B) ETVAX vaccine (n = 15) or (C) ETVAX with 10 µg dmLT adjuvant (n = 15). Participants were not allowed to eat or drink one hour before and after treatment.

### 2.4 Follow-up for adverse reactions

Study participants were given memory aids to record solicited symptoms (e.g. nausea, vomiting, diarrhea, loose stools, abdominal pain and fever). Both solicited and unsolicited adverse events (AEs) were assessed by trained study staff by carrying out active surveillance by home visits each day for 7 days after each vaccination and again on day 28 ± 2 and 182 ± 14 after the first vaccination. Clinical chemistry and hematology tests were performed at screening and on days 7 ± 1 after the first immunization, physical examinations were performed at screening and on day 19 + 1 and 42 ± 4 after the first immunization. Safety in adults was evaluated until day 3 after the second dose by an independent protocol safety team and a data safety monitoring committee before vaccinations in descending age groups of children were initiated.

### 2.5 Measurement of immune responses

Heparinized venous blood samples were obtained prior to the first immunization (day −7 to −4), on day 7 ± 1 after the first dose and on day 19 (5 days after the 2nd dose); i.e. optimal time points for assessment of circulating ASCs after oral vaccination [26]. Mucosal immune responses were evaluated by measuring vaccine specific IgA antibodies in ALS specimens and systemic immune responses by measuring IgA and IgG antibodies levels in plasma [14], [15]. PBMCs and plasma were separated from the blood by density-gradient centrifugation on Ficoll-Isopaque (Pharmacia, Uppsala, Sweden). Plasma specimens were preserved at −20 °C. For ALS preparation, 10^7^ PBMCs/ml were cultured in 96-well plates (200 µl/well) for 48 h at 37 °C with 5% CO_2_, supernatants were collected and stored at −70 °C [28].

IgA antibody levels in ALS specimens were analyzed by a novel ECL assay established for all five primary vaccine antigens (CFA/I, CS3, CS5, CS6 and LTB). The optimization of the new ECL assay was performed using samples collected from previous trials of ETVAX in Sweden [16], [18] and included titration of antigen coating concentrations, testing of coating buffer compositions, incubation times and temperatures. The optimized protocol was then used to measure IgA responses in ALS specimens from Bangladeshi adults. Standard binding MULTI ARRAY 96-well plates (MSD) were coated with 0.5 µg/ml of CF or LTB (without GM1) in carbonate buffer (pH 9.8) at +4 °C overnight, after 10 min shaking at room temperature. Plates were blocked (1% casein in PBS, Thermo Fisher, 1 h) and ALS samples diluted 1 to 5 in 1% casein-PBS were added (25 µl/well, single wells) for 2 h. IgA antibodies were detected using anti-Human NHP IgA Sulfo Tag antibodies (1 µg/ml, MSD, 1 h). Read Buffer T (1X concentration, MSD) was added and reactions were immediately analyzed on a Meso Quickplex SQ 120 reader (MSD). Plates were washed after each step using 0.05% Tween PBS. Incubations were performed at room temperature with shaking. Plasma and also ALS samples were analyzed by ELISA using plates coated with CFA/I, CS3, CS5, CS6 and GM1 plus LTB [15], [16]. The recombinant CFA/I antigen used in the ELISA and ECL assays was prepared from a rough *E. coli* strain at the University of Gothenburg. The CS3 and CS5 antigens were prepared from O139 LPS *E. coli* strains at Scandinavian Biopharma, Sweden. The CS6 antigen was a kind gift from F. Cassels, USA. LTB was produced at Scandinavian Biopharma. The CFA/I, CS6 and LTB antigens were completely LPS free. Silver staining indicated some LPS (approximately 40 µg LPS per mg protein) in the CS3 but not in the CS5 antigen. For control experiments, a CS3 antigen provided by NIH, USA (now available through BEI Resources NR-49113), containing only trace amounts of LPS (1 EU per mg of protein, corresponding to 100 pg LPS per mg of protein), was used. Repeat analyses on about 20% of samples were conducted for control purposes.

Stool samples were collected prior to the first immunization (day −7 to −4) and day 7, 19, and 28 post immunization and extracted, stored at −70 °C and analyzed as previously described [22], [26], [37], [38]. However, a majority of the samples contained low and variable levels of total SIgA, and did not meet the inclusion criteria for analysis [13], [16], [38]. Therefore, analysis of CF and LTB specific antibody responses were not evaluated in fecal specimens from adults.

### 2.6 Statistical analysis

The sample size was selected to detect frequent AEs. Given a sample size of 15 adults, and subsequently the same sample size in toddlers and younger children each receiving one of varying dose levels of ETVAX with and without dmLT, the study would have an approximately 80% and 90% chance of observing at least one serious AE (SAE) or AE of special interest for events that occur at a rate of 10.3% and 14.3%, respectively. Additionally, if no SAEs are observed in 15 participants, the upper bound of the one-sided 95% confidence interval on the rate of SAE occurrence is approximately 18%.

All results were log10 transformed. The magnitudes of immune responses (fold rises) were calculated as the post-immunization divided by pre-immunization antibody levels and twofold increases were regarded as responses [16]. Comparisons of pre- and post-levels within groups were evaluated using a paired *t*-test and responses between different groups using an unpaired *t*-test. Responder frequencies were evaluated using Fisher exact test. The Pearson correlation coefficient was used to measure the correlations. *P*-values < 0.05 were considered as significant. All statistical analyses were performed with GraphPad Prism version 5.0.

## 3 Results

### 3.1 Study participants

**Fig. 1:**
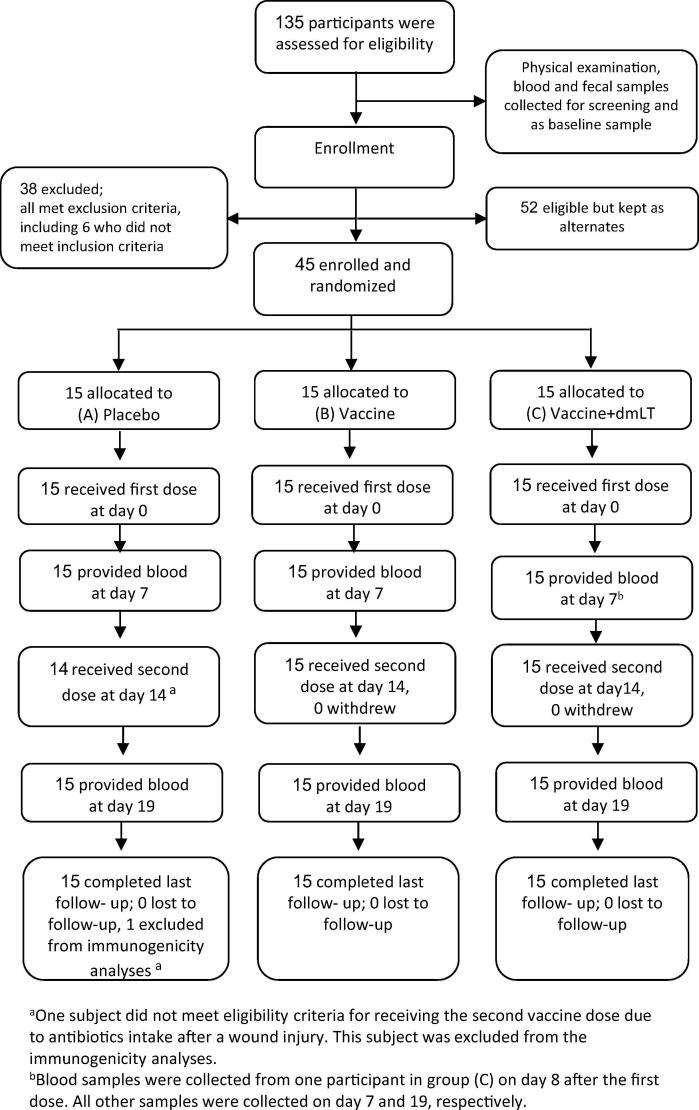
Trial profile.

Forty five healthy adults were enrolled, from 135 participants screened, and were randomized into the three treatment groups (groups A-C; Fig. 1). The age distributions were comparable among the groups but more females than males were enrolled in all groups (Table 1).

**Table 1:** Participant demographics (safety analysis set).

	(A) Placebo (n = 15)	(B) Vaccine (n = 15)	(C) Vaccine + dmLT (n = 15)	Total (n = 45)
*Age (years)*				
Mean (SD)	31.5 (5.6)	30.1 (7.0)	29.4 (7.0)	30.3 (6.4)
Range	20–40	19–44	20–41	19–44

*Gender (no and freq. of participants)*				
Female	10 (66.7%)	8 (53.3%)	11 (73.3%)	29 (64.4%)
Male	5 (33.3%)	7 (46.7%)	4 (26.7%)	16 (35.6%)

### 3.2 Adverse events and safety

ETVAX administered alone or in combination with 10 µg dmLT was safe and well tolerated in all adults with only two solicited AEs and no SAEs reported at any time point during the study period (Table 2).

**Table 2:** Solicited AEs in study participants after one or two vaccine doses (safety analysis set).

	(A) Placebo (n = 15)	(B) Vaccine (n = 15)	(C) Vaccine + dmLT (n = 15)
Nausea	0	0	0
Vomiting	0	0	0
Diarrhea	0	0	0
Loose Stools	0	0	0
Abdominal Pain	0	0	0
Fever	1 (6.7%)^a^	0	1 (6.7%)^b^
Total	1 (6.7%)	0	1 (6.7%)

a Mild fever appeared and resolved spontaneously on the same day of receiving placebo.

b Mild fever appeared two days after receiving the first vaccine dose and resolved one day later.

### 3.3 Immunogenicity

#### 3.3.1 Correlations between results from ECL and ELISA assays

**Fig. 2:**
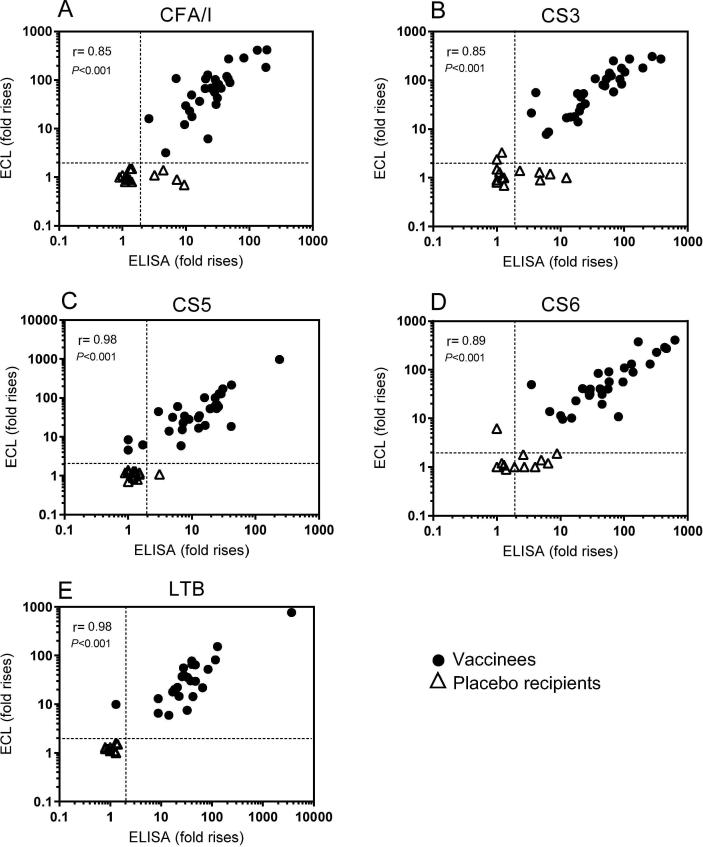
Comparison of ALS results obtained using the novel ECL and the traditional ELISA assays. Magnitudes of responses (fold rises) of specific IgA antibodies against (A) CFA/I, (B) CS3, (C) CS5, (D) CS6 and (E) LTB antigens were analyzed in day 19 ALS specimens (n = 30 vaccinees and n = 14 placebo recipients). Dashed lines indicate fold rises ≥2.

To determine if an ECL assay could be used to measure ASC responses using low antibody concentrations in small volumes of lymphocyte secretions, IgA levels in ALS samples were measured in parallel by ELISA and the novel ECL assay. High correlations between the magnitudes of ALS responses (fold rises in antibody levels in post- compared to pre-vaccination samples) determined by the optimized ECL and the traditional ELISA assays were found for all five primary antigens using samples collected 7 days after the first dose (r = 0.88–0.96, *P* < 0.001, data not shown) as well as 5 days after the second dose (r = 0.85-0.98, *P* < 0.001, Fig. 2). Magnitudes of responses to CFA/I and CS5 were 3- to 4-fold higher determined by ECL compared to ELISA, but still correlated very well, whereas the magnitudes of responses to CS3, CS6 and LTB were comparable in the two assays. Considering the excellent performance of the ECL assay and the relatively small volumes of ALS samples available from some participants, the ECL assay was selected as the primary readout for the ALS responses. Unblinding of the results revealed that the vast majority of placebo recipients had responses to all antigens below the 2-fold cut-off for positivity in both assays (Fig. 2). However, the discrimination between vaccinees and placebo recipients was more distinct using the ECL assay, since a few more placebo recipients showed weak ALS responses to CFs in ELISA (Fig. 2).

#### 3.3.2 Intestine derived IgA ALS responses

**Fig. 3:**
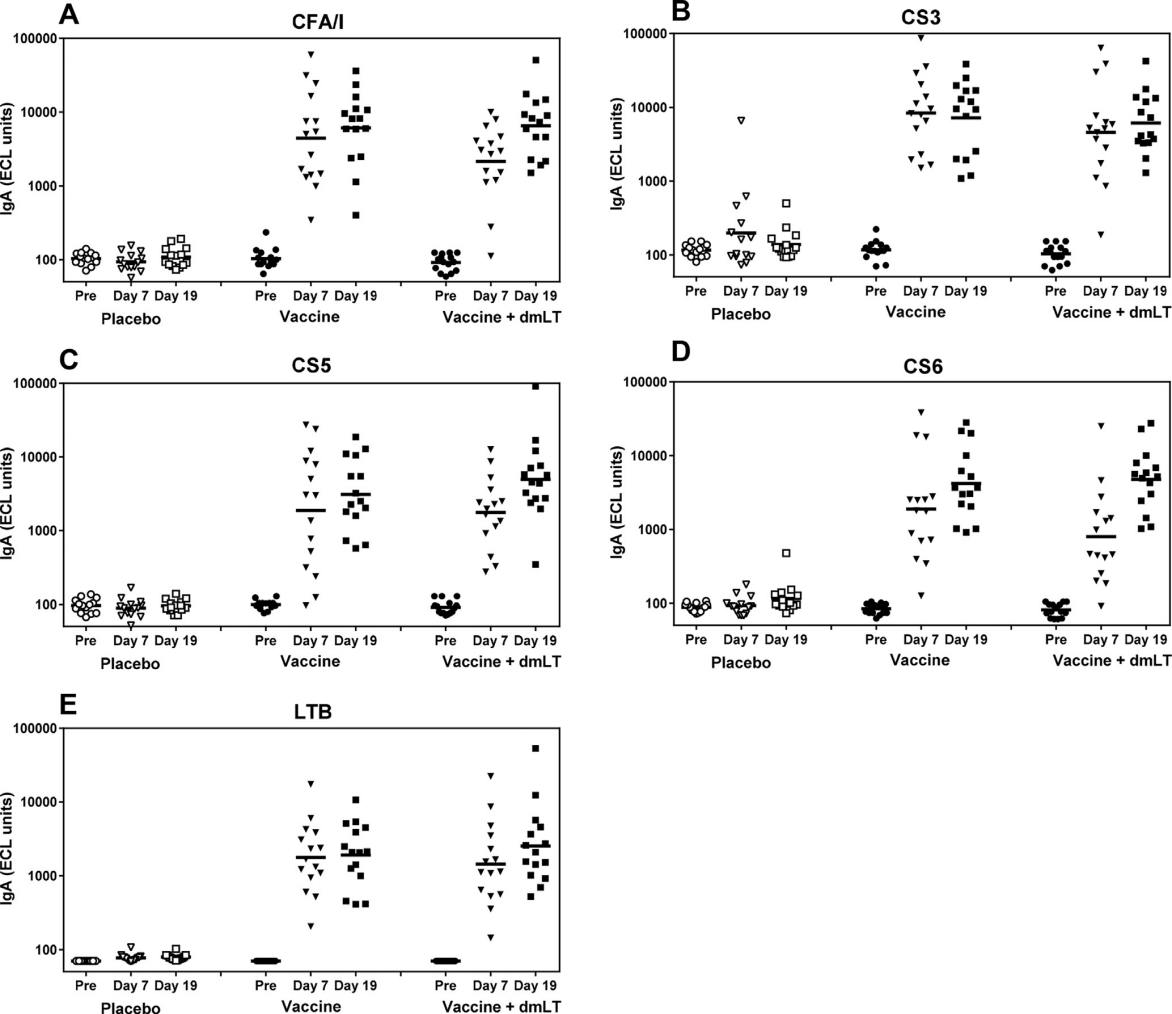
ALS IgA responses in participants receiving placebo (n = 14), vaccine (n = 15) and vaccine plus dmLT (n = 15). Levels of IgA antibodies specific for (A) CFA/I, (B) CS3, (C) CS5, (D) CS6 and (E) LTB were analyzed using the ECL assay 4–7 days before administration of the first treatment dose (Pre), 7 days after the first dose (Day 7) and 5 days after the second dose (Day 19), respectively. Each symbol represents one subject and horizontal lines indicate geometric means.

ECL analysis of ALS specimens showed that ETVAX alone or with dmLT adjuvant elicited significant increases of specific IgA antibody responses against all four vaccine CFs and LTB (Fig. 3 and Table 3). The magnitudes of responses were high already 7 days after the first vaccine dose compared to pre-immunization levels and responses remained at similar levels (anti-CS3 and anti-LTB responses) or increased further 5 days (day 19) after the second dose (*P* < 0.001 for comparisons of magnitudes of ALS responses on day 7 or day 19 versus pre-vaccination levels for all antigens). Responder frequencies among the vaccinees were 90–100% against all antigens already after the first dose and 100% after the second dose (Table 3). In contrast, very few placebo recipients responded to any of the antigens, and then only to CS3 (4/14 subjects) or CS6 (1/14). Both the magnitudes of responses and frequencies of responders were significantly higher (*P* < 0.001) in the vaccinated groups compared to the placebo group on both day 7 and 19 (Table 3).

**Table 3:** Magnitudes^a^ and frequencies^b^ of ALS IgA responses against the five primary vaccine antigens determined by ECL after administration of one and two treatment doses (per protocol analysis set).

	Dose 1^c^			Dose 2^c^		
	Placebo	Vaccine	Vaccine + dmLT	Placebo	Vaccine	Vaccine + dmLT
*CFA/I*						
GM	0.9	42.8	23.4	1.0	52.7	71.1
RF	0/14 (0%)	15/15 (100%)	14/15 (93.3%)	0/14 (0%)	15/15 (100%)	15/15 (100%)

*CS3*						
GM	1.7	71.9	44.7	1.2	61.8	59.8
RF	4/14 (28.6%)^d^	15/15 (100%)	14/15 (93.3%)	2/14 (14.3%)^d^	15/15 (100%)	15/15 (100%)

*CS5*						
GM	0.9	19.0	19.4	1.0	31.4	54.3
RF	0/14 (0%)	13/15 (86.7%)	15/15 (100%)	0/14 (0%)	15/15 (100%)	15/15 (100%)

*CS6*						
GM	1.1	22.4	9.9	1.3	49.7	59.1
RF	0/14 (0%)	14/15 (93.3%)	13/15 (86.7%)	1/14 (7.1%)	15/15 (100%)	15/15 (100%)

*LTB*						
GM	1.1	25.3	20.6	1.1	27.3	36.0
RF	0/14 (0%)	15/15 (100%)	15/15 (100%)	0/14 (0%)	15/15 (100%)	15/15 (100%)

a Magnitudes of responses were expressed as geometric mean (GM) of fold rises.

b Fold rises ≥2 were considered as responses [16] and responder frequencies (RF) using this cut-off are indicated.

c Magnitudes and responder frequencies were significantly higher (*P* < 0.001) in the vaccine and vaccine + dmLT groups, respectively, compared to the placebo group.

d Only 1/14 placebo recipients (7%) showed responses to CS3 after dose 1 as well as dose 2 when evaluated using a CS3 antigen preparation containing only trace amounts of LPS (100 pg per mg of protein), whereas vaccinees had comparable responses to both CS3 preparations.

Addition of dmLT to the vaccine did not have any significant impact on the ALS responses. Magnitudes and frequencies of responses were similar both on day 7 and day 19 in the vaccine compared to the vaccine + dmLT groups (Fig. 3 and Table 3). The ALS responder frequencies recorded using ELISA and ECL assays were similar in the vaccine groups, but slightly higher frequencies of responders were recorded in the placebo group using ELISA (Table 4).

**Table 4:** Magnitudes^a^ and frequencies^b^ of ALS IgA responses against the five primary vaccine antigens determined by ELISA after administration of one and two treatment doses (per protocol analysis set).

	Dose 1^c^			Dose 2^c^		
	Placebo	Vaccine	Vaccine + dmLT	Placebo	Vaccine	Vaccine + dmLT
*CFA/I*						
GM	1.0	17.1	10.1	1.6	27.5	23.6
RF	0/14 (0%)	14/15 (93.3%)	13/15 (86.7%)	4/14(28.6%)	15/15 (100%)	15/15 (100%)

*CS3*						
GM	1.5	55.0	17.8	1.9	46.9	27.3
RF	3/14 (21.4%)	15/15 (100%)	12/15 (80%)	5/14 (35.7%)	15/15 (100%)	15/15 (100%)

*CS5*						
GM	1.0	5.3	5.6	1.1	9.1	15.2
RF	0/14 (0%)	7/13 (53.8%)	11/15 (73.3)	1/14 (7.1%)	12/14 (85.7%)	14/15 (93.3%)

*CS6*						
GM	1.2	19.0	6.2	2.0	65.7	43.3
RF	1/14 (7.1%)	13/15 (86.7%)	9/15 (60%)	6/14 (42.9%)	15/15 (100%)	15/15 (100%)

*LTB*						
GM	1.0	21.2	23.6	1.0	30.5	38.9
RF	0/11 (0%)	11/12 (91.7%)	11/13 (84.6%)	0/11 (0%)	12/12 (100%)	11/12 (91.7%)

a Magnitudes of responses were expressed as geometric mean (GM) of fold rises.

b Fold rises ≥2 were considered as responses [16] and responder frequencies (RF) using this cut-off are indicated.

c Magnitudes and responder frequencies were significantly higher (*P* < 0.001) in the vaccine and vaccine + dmLT groups, respectively, compared to the placebo group.

Since weak CF responses were detected among a few placebo recipients, all antigens used for the immunological analyses were tested for possible LPS contamination by sensitive silver staining techniques. These analyses indicated some LPS contamination in the CS3 antigen (approximately 40 µg per mg protein), but not in any of the other antigens. Therefore, control analyses using a CS3 antigen preparation that contained only trace amounts of LPS (100 pg per mg of protein) provided by NIH were performed. Only one of the four placebo recipients responding to the original CS3 preparation prepared from an *E. coli* strain expressing O139 LPS showed responses to the low LPS CS3 antigen when tested by ECL (Table 3). In contrast, when ALS samples from vaccine recipients were retested using the low LPS CS3, including samples from all weak CS3 responders and a subset of medium or strong responders, comparable ALS responses to both CS3 preparations were recorded.

#### 3.3.3 Plasma antibody responses

**Fig. 4:**
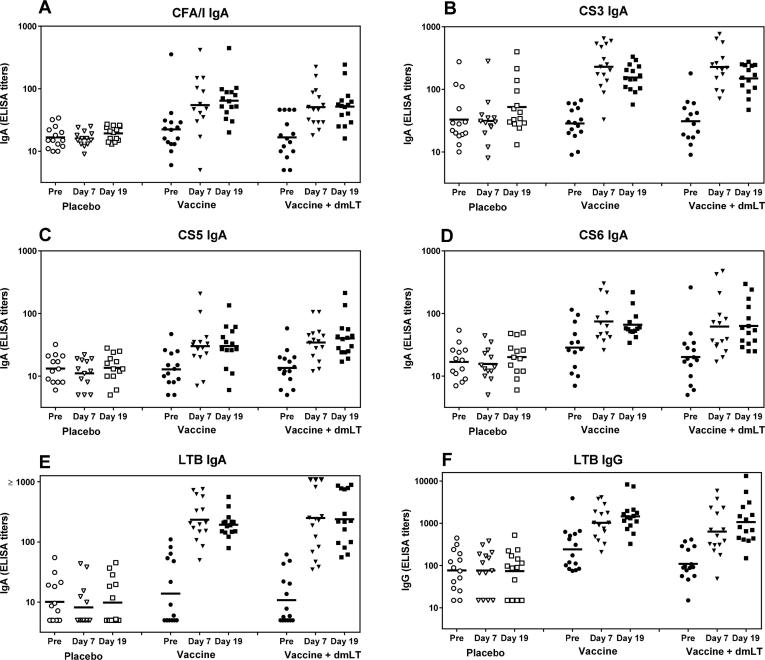
Plasma IgA and IgG responses in participants receiving placebo (n = 13–14), vaccine (n = 13–15) and vaccine plus dmLT (n = 14–15). Titers of IgA antibodies specific for (A) CFA/I, (B) CS3, (C) CS5, (D) CS6, (E) LTB and (F) IgG antibodies specific for LTB were analyzed using ELISA. Samples were collected 4–7 days before administration of the first treatment dose (Pre), 7 days after the first dose (Day 7) and 5 days after the second dose (Day 19), respectively. Each symbol represents one subject and horizontal lines indicate geometric means. Limited sample volumes precluded analysis of plasma responses in a few participants (1–2 per group).

CF specific IgA and LTB specific IgA and IgG responses were measured in plasma using ELISA. Significant plasma responses were found 7 days after the first dose and the magnitudes of responses against all vaccine antigens remained at comparable levels 5 days after the second vaccination (*P* < 0.001 for comparisons of plasma response magnitudes on day 7 or day 19 versus pre-vaccination IgA levels; Fig. 4 and Table 5). Highest and consistently most frequent IgA responses in all groups were observed against LTB (100% after both doses) and CS3 (about 90%), whereas 60–93% of subjects responded to CFA/I, CS5 and CS6 (Table 5). In contrast, very few participants responded in the placebo group to any of the antigens, except CS3 (3/14 subjects) and LTB (1/14 and 2/14 subjects for IgA and IgG, respectively). Both the magnitudes and frequencies of responders were significantly higher in the vaccine groups compared to the placebo group after both treatment doses (*P* < 0.001). All weak CS3 plasma responses among both placebo and vaccine recipients were confirmed using the low LPS CS3 preparation (Table 5).

**Table 5:** Magnitudes^a^ and frequencies^b^ of plasma IgA and IgG responses against the five primary vaccine antigens determined by ELISA after administration of one and two treatment doses (per protocol analysis set).

	Dose 1^c^			Dose 2^c^		
	Placebo	Vaccine	Vaccine + dmLT	Placebo	Vaccine	Vaccine + dmLT
*CFA/I IgA*						
GM	1.0	2.1	3.1	1.2	2.9	3.1
RF	0/13 (0%)	7/13 (53.8%)	11/15 (73.3%)	0/14 (0%)	11/15 (73.3%)	12/15 (80.0%)

*CS3 IgA*						
GM	1.1	8.0	7.3	1.6	5.4	4.8
RF	2/13 (15.4%)^d^	13/15 (86.7%)	13/14 (92.9%)	3/14 (21.4%)^d^	14/15 (93.3%)	12/14 (85.7%)

*CS5 IgA*						
GM	0.8	2.3	2.5	1.0	2.4	3.0
RF	0/13 (0%)	9/14 (64.3%)	10/15 (66.7%)	0/13 (0%)	7/14 (50.0%)	12/15 (80.0%)

*CS6 IgA*						
GM	0.9	2.6	3.1	1.2	2.3	3.1
RF	0/13 (0%)	6/13 (46.2%)	10/15 (66.7%)	0/13 (0%)	8/13 (61.5%)	11/15 (73.3%)

*LTB IgA*						
GM	0.8	16.8	23.1	1.0	13.9	22.0
RF	0/14 (0%)	15/15 (100%)	15/15 (100%)	1/14 (7.1%)	15/15 (100%)	15/15 (100%)

*LTB IgG*						
GM	1.0	4.2	5.8	1.0	6.1	9.8
RF	1/14 (7.1%)	11/15 (73.3%)	13/15 (86.7%)	2/14 (14.3%)	14/15 (93.3%)	13/15 (86.7%)

a Magnitudes of responses were expressed as geometric mean (GM) of fold rises.

b Fold rises ≥2 were considered as responses [16] and responder frequencies (RF) using this cut-off are indicated.

c Magnitudes and responder frequencies were significantly higher (*P* < 0.001) in the vaccine and vaccine + dmLT groups, respectively, compared to the placebo group.

d Comparable CS3 responses were observed using a CS3 antigen preparation containing only trace amounts of LPS (100 pg per mg of protein) among both placebo and vaccine recipients.

Addition of dmLT to the vaccine did not cause any significant effect on plasma antibody responses. However, there was a trend for higher (10–30%) responder frequencies against CFA/I (dose 1), CS5 (dose 2) and CS6 (both doses) in participants receiving ETVAX + dmLT compared to ETVAX alone (Table 5, *P* > 0.05). A similar trend was also apparent when responses to multiple antigens were considered together; 93% of the participants immunized with ETVAX + dmLT and 62% with the vaccine alone responded to ≥4 antigens and 57% (ETVAX + dmLT) versus 38% (ETVAX) to all 5 antigens, respectively (Table 6). The breadth of the antigenic response at less stringent levels (≤ 3 antigens) were essentially identical between subjects receiving the vaccine alone or with dmLT.

**Table 6:** Frequencies of IgA responders against different numbers (1–5) of primary vaccine antigens in plasma after one or two doses.

Frequency of subjects^a^ responding to	Placebo	Vaccine^c^	Vaccine + dmLTc,^d^
5 antigens^b^	0/13 (0%)	5/13 (38%)	8/14 (57%)
≥4 antigens	0/13 (0%)	8/13 (62%)	13/14 (93%)
≥3 antigens	0/13 (0%)	12/13 (92%)	13/14 (93%)
≥2 antigens	1/13 (8%)	12/13 (92%)	13/14 (93%)
≥1 antigens	3/13 (20%)	13/13 (100%)	14/14 (100%)
0 antigens	10/13 (77%)	0/13 (0%)	0/14 (0%)

a Subjects from whom plasma specimens were available for analysis of responses to all five antigens.

b LTB, CFA/I, CS3, CS5, CS6.

c Responder frequencies against ≥1–5 antigens were significantly higher (*P* < 0.05) in the vaccine and vaccine + dmLT groups, respectively, compared to the placebo group.

d Responder frequencies were not significantly different (*P* > 0.05) in the vaccine compared to the vaccine + dmLT group. However, including dmLT in the vaccine formulation appeared to favor a broader antigenic response than that achieved with the vaccine alone, particularly when plasma IgA response frequencies to ≥4 or 5 antigens were considered.

## 4 Discussion

This is the first clinical trial of the second generation oral inactivated multivalent ETEC vaccine ETVAX conducted in an ETEC endemic country. We show that two full doses of ETVAX, both when administered alone and with 10 µg dmLT adjuvant, were safe and well tolerated in the healthy Bangladeshi adults tested, confirming previous safety data from studies in Swedish adults [15], [16], [18]. We further demonstrate that the vaccine is highly immunogenic in Bangladeshi adults, inducing intestine-derived ASC responses as detected by the ALS method in all, and plasma antibody responses in a majority, of the vaccinees to all primary vaccine antigens (four CFs and LTB). We also show that the ALS results obtained using a novel ECL assay and traditional ELISA correlated very well for all vaccine CFs and LTB. Since the ECL assay using the MSD platform only requires 5 µl of ALS sample compared to 75 µl in ELISA for determination of responses to each antigen, only 150 µl of sample (collected from a single ALS well on a 96-well plate, containing 2x10^6^ PBMCs derived from 1 to 2 ml blood) can be used for at least 30 ECL analyses compared to two ELISA tests. Thus, the ECL assay is highly useful for future assessment of intestine-derived IgA ALS responses in pediatric studies, and also in studies of ASC responses to multivalent vaccines. Furthermore, the wide dynamic range of the ECL assay made it possible to use a single sample dilution, while sample titration is normally used in ELISA. Given the favorable results, we selected the ECL assay as the primary method for assessing ALS responses both in adults and subsequently in children in the trial. Slightly higher rates of weak antibody responses were seen in the placebo group using ELISA compared to ECL, indicating that the ECL assay may also be more specific.

Evaluation of intestine-derived ALS responses using ECL showed that 90–100% of the vaccinees responded to all primary vaccine antigens after a single dose and 100% after the second dose, even in the absence of dmLT adjuvant. These responses were clearly more frequent than in Swedish vaccinees; ALS responses after the first dose were infrequent in the Swedes and 60–70% responded against CFA/I, CS5 and CS6 and 80–100% against LTB and CS3 after the second dose [16]. Our finding that almost all Bangladeshis responded strongly already after the first dose and that very few showed enhanced responses after the second dose support development of anamnestic responses in the Bangladeshi volunteers, since we have previously shown that only primed subjects respond strongly to a single dose of ETVAX [18]. The results are also consistent with the high prevalence of ETEC infection in Bangladesh. During the first two years of life, 20% of all diarrheal cases at the trial site in Mirpur in urban Dhaka have been shown to be due to ETEC infection, with an incidence of 0.5 episode/child/year [2] and ETEC is also an important diarrheal pathogen in Bangladeshi adults [39]. Analysis of the relative distribution of CFs in ETEC isolates from diarrhea cases at the icddr,b hospital between year 2007 and 2012 suggests that the predominant CFs on ETEC isolated from diarrhea cases are CS5 + CS6, CFA/I, CS7, CS17, CS1 + CS3, CS6 and CS14 [40]. Thus, since ETEC infection with strains expressing vaccine CFs and related CFs are common in the study population, we postulate that almost all Bangladeshi adults have been naturally primed with vaccine related ETEC strains in this study, thus explaining the high and frequent responses already after the first vaccine dose.

In this study, comparable or even slightly higher ASC/ALS responses were found against most vaccine antigens 5 days after the second compared to 7 days after the first vaccine dose. This is in contrast to previous studies of ETEC and cholera vaccines, where lower ASC responses were usually found after the second dose [10], [41], [42]. However, in most of these earlier studies blood samples were collected 7 days after the second immunization, which seems to be too late to capture maximal ASC responses, and this has most likely resulted in underestimation of ASC responses in previous trials [11], [23], [43], [44]. We have recently shown, using a model oral cholera vaccine, Dukoral, that ASC/ALS responses peak 5 rather than 7 days after a second or late booster vaccination both in children and adults in Bangladesh [26], therefore different and more optimal time points were selected for sampling in the present study.

Plasma antibody responses against CFs and LTB were also found to be high in this endemic setting. In analogy with findings in adult Swedes almost all the Bangladeshi vaccinees responded with plasma IgA (100%) and IgG (73–93%) anti-LTB responses after either dose of ETVAX whereas anti-CF responses were considerably more frequent in the Bangladeshi than the Swedish adults (60–90% vs. 3–19%) [16]. The comparatively high pre-vaccination plasma titers recorded in several of the Bangladeshi vaccinees is consistent with previous natural ETEC priming [45], [46]. Our results suggest that numerous ETEC infections may prime the systemic CF-specific B cells more efficiently than vaccination, since Swedish adults had weak serum responses to CFs also after receiving a late booster dose (third dose) of ETVAX [18].

A few placebo recipients responded against CS3 (14–28%), CS6 (0–7%) and LTB (0–14%) in plasma and/or ALS, as assessed by ECL and ELISA, respectively, but most responses were of low magnitudes and responses were mostly recorded against a single antigen and only in one type of specimen for each subject. The weak responses may be due to contamination (e.g. by LPS) in the antigen preparations. This is supported by our observation that only one of the four placebo recipients showed ALS responses to a low LPS CS3 preparation, whereas all tested vaccinees responded with comparable magnitudes to this CS3 antigen. The other antigens used in the ELISA and ECL assays were produced from LPS negative rough strains or purified to a higher degree than the CS3 antigen. These findings support the importance of using highly purified antigens for sensitive immunological analyses. However, plasma responses to CS3 were confirmed in placebo recipients using the low LPS antigen, suggesting that responses among placebo recipients may also be due to infection with ETEC, cholera or other bacteria expressing homologous or related antigens during the study period.

The first generation ETEC vaccine induced ASC and plasma responses against CFs and CTB in a majority of adult Bangladeshi vaccinees [10]. However, a direct comparison with the present study could not be done since different methods for monitoring immune responses have been used, i.e. ALS instead of ELISPOT, use of optimal time points for assessing ASC/ALS responses and more pure CF antigen preparations than were previously available. The only relevant comparison between immune responses induced by the first and the second generation vaccines is probably of plasma responses to the toxoid component of the vaccines, since the methods for evaluation of such responses have changed very little and time points for assessment of plasma responses are not as critical. The LCTB*A* toxoid, which is a hybrid between LTB and CTB [36], induced strong and frequent (100%) plasma IgA responses against LTB which were similar or even slightly more frequent than IgA responses against CTB after vaccination with the first generation rCTB-CF ETEC vaccine or the CTB-containing Dukoral vaccine [10], [11], [26]. Furthermore, a previous study in Sweden has shown that significantly higher systemic responses to LTB were induced by vaccination with LCTB*A* compared to vaccination with CTB in the same trial and that antibodies induced by LCTB*A* also react efficiently with CTB [15]. Thus, the LCTB*A* toxoid may induce protection against both LT-ETEC and cholera in endemic settings.

The systemic responses were measured by ELISA in the present study, since larger volumes of plasma compared to ALS samples were available. However, we have recently shown that plasma IgA and IgG responses to ETEC CFs and LTB can be efficiently evaluated by the ECL method (Svennerholm et al., unpublished data). MSD ECL assays have previously been used to evaluate serum IgG antibody responses to licensed parenteral vaccines or vaccine candidates, e.g. vaccines against *Streptococcus pneumoniae*, human papilloma virus, respiratory syncytial virus and rabies, utilizing carbohydrates, proteins or virus like particles for coating in both single and multiplex assays [32], [33], [34], [35], [47]. However, to our knowledge, this is the first study where the MSD ECL technology has been used to successfully evaluate intestine-derived IgA antibody responses induced by an oral vaccine. In the future, the possibility of establishing a multiplex ECL method including several ETEC antigens in the same microtiter well may be considered to further reduce sample volumes and working time.

We also attempted to measure mucosal IgA responses more directly, in fecal extracts. However, since fecal extracts from the Bangladeshi adults contained low and very variable levels of total SIgA and did not fulfill the assay criteria for specimen inclusion, such analyses were not meaningful. Similar low and variable SIgA levels were observed in adult Bangladeshis in a recent cholera vaccine study [26]. In contrast, fecal samples from Swedish adults extracted using the same method contained higher SIgA levels and robust SIgA responses were recorded against all primary vaccine antigens after ETVAX vaccination [16]. The reason for this discrepancy may be due to several factors, including different diets and microbiota. Previous analyses of vaccine induced immune responses in fecal samples from Bangladeshi adults also resulted in higher IgA levels [10], [22], [23], but total IgA rather than total SIgA was measured in these studies and some IgA transudated from serum may thus have been detected. Importantly, preliminary data from infants participating in the ETVAX trial in Bangladesh, as well as previous data from children vaccinated with ETEC or cholera vaccines, support that fecal samples from Bangladeshi infants contain higher and more stable levels of SIgA than adult samples and therefore are more suitable for immunogenicity analyses [10], [11], [26].

Addition of dmLT adjuvant to the vaccine had no apparent effect on the ALS responses in the adults in this study. In contrast, in adult Swedes, 10 µg of dmLT significantly enhanced ASC responses to CS6, which is the CF present in the lowest amount in the vaccine [16]. However, a higher dose of dmLT, 25 µg, had no significant influence on ALS responses, and hence 10 µg dmLT was tested in the Bangladeshi adults [16]. The reason why dmLT did not induce a similar enhancement of the ALS responses in this trial may be explained by extensive natural priming of the Bangladeshis, limiting the ability of dmLT to further enhance the already strong ALS responses. However, a trend (not statistically significant) for an adjuvant effect was observed for plasma responses in the Bangladeshis against the CFs present in the lowest amounts in the vaccine (CS5 and CS6), consistent with the dose-sparing effect of dmLT previously observed in ETVAX vaccinated mice [14]. The potential effect of dmLT when co-administered with a lower dose of vaccine will be evaluated in continued studies in children and infants. A strong trend toward an adjuvant effect was also noted when the antigenic breadth of the plasma IgA response was evaluated, particularly when responses to 4 or more or 5 vaccine antigens were considered. This apparent impact of dmLT on the breadth of the vaccine-specific antibody responses will also be evaluated more fully as lower vaccine doses are given in follow-on studies in children and infants.

Overall, this trial demonstrates that the oral ETVAX vaccine is safe in adults and induces strong mucosal as well as systemic immune responses against key vaccine antigens. These findings have provided a base for further evaluation of this vaccine in descending age groups in children and infants. The ECL assay established here allows sensitive and specific analysis of ALS responses to all key vaccine components even in young children and infants.
